# Supplementary material for: Empirical methods for controlling false positives and estimating confidence in ChIP-Seq peaks
Source: BMC Bioinformatics. 2008 Dec 5;9:523. doi: 10.1186/1471-2105-9-523 (PMC2628906; doi:10.1186/1471-2105-9-523)
Supplement: Additional File 1 — A variety of html documents from the USeq web site detailing the available applications, their best usage, output file type descriptions, command line menus, etc. [file 1471-2105-9-523-S1.zip › USeqUserGuides/JavaDoc.zip!/JavaDoc/edu/utah/seq/parsers/ElandSequenceParser.html]

ElandSequenceParser


|  |  |  |
| --- | --- | --- |
| **Overview** \| **Package** \| **Class** \| **Use** \| **Tree** \| **Deprecated** \| **Index** \| **Help** | |  |
| **PREV CLASS**   **NEXT CLASS** | **FRAMES**    **NO FRAMES**     **All Classes** |  |
| SUMMARY: NESTED \| FIELD \| CONSTR \| METHOD | DETAIL: FIELD \| CONSTR \| METHOD |  |


---


## edu.utah.seq.parsers Class ElandSequenceParser

```
java.lang.Object
  edu.utah.seq.parsers.ElandSequenceParser
```

---

``` public class ElandSequenceParser extends java.lang.Object ```

Parses an Eland Extended xxx.export.txt or xxx.sorted.txt for sequencing information.
Generates track data representing the sum of the base quality scores for every position, GATC
as well as a consensus track (1-fraction consensus)
Positions are in interbase coordinates (0 start, end excluded).

**Author:**
:   david.nix@hci.utah.edu

---

**Constructor Summary**

`ElandSequenceParser(java.lang.String[] args)`


| **Method Summary** | |
| --- | --- |
| `static java.lang.String` | `formatFraction(int number, int total, int numberDecimals)`             Returns number space (percent%) |
| `static void` | `main(java.lang.String[] args)` |
| `void` | `parseChromData(java.lang.String chromosome, int[][] gatc)` |
| `void` | `parseChromFile(java.io.File file, int[][] gatc)` |
| `static void` | `printDocs()` |
| `void` | `processArgs(java.lang.String[] args)`             This method will process each argument and assign new varibles |
| `float[]` | `scoreConsensus(int[][] gatc, java.lang.String seq, float minTotalToScore)` |
| `void` | `splitByChromosome(java.io.File dataFile)` |
| `void` | `writeConsensusBars(java.lang.String chrom, float[] frac)` |
| `void` | `writeGATCBars(java.lang.String chrom, int[][] gatc)` |

**Methods inherited from class java.lang.Object**

`equals, getClass, hashCode, notify, notifyAll, toString, wait, wait, wait`

**Constructor Detail**

### ElandSequenceParser

```
public ElandSequenceParser(java.lang.String[] args)
```


**Method Detail**

### scoreConsensus

```
public float[] scoreConsensus(int[][] gatc,
                              java.lang.String seq,
                              float minTotalToScore)
```

---


### parseChromData

```
public void parseChromData(java.lang.String chromosome,
                           int[][] gatc)
```

---


### parseChromFile

```
public void parseChromFile(java.io.File file,
                           int[][] gatc)
```

---


### formatFraction

```
public static java.lang.String formatFraction(int number,
                                              int total,
                                              int numberDecimals)
```

:   Returns number space (percent%)

---


### splitByChromosome

```
public void splitByChromosome(java.io.File dataFile)
```

---


### writeConsensusBars

```
public void writeConsensusBars(java.lang.String chrom,
                               float[] frac)
```

---


### writeGATCBars

```
public void writeGATCBars(java.lang.String chrom,
                          int[][] gatc)
```

---


### main

```
public static void main(java.lang.String[] args)
```

---


### processArgs

```
public void processArgs(java.lang.String[] args)
```

:   This method will process each argument and assign new varibles

---


### printDocs

```
public static void printDocs()
```


---


|  |  |  |
| --- | --- | --- |
| **Overview** \| **Package** \| **Class** \| **Use** \| **Tree** \| **Deprecated** \| **Index** \| **Help** | |  |
| **PREV CLASS**   **NEXT CLASS** | **FRAMES**    **NO FRAMES**     **All Classes** |  |
| SUMMARY: NESTED \| FIELD \| CONSTR \| METHOD | DETAIL: FIELD \| CONSTR \| METHOD |  |


---
